# Women’s Lived Experiences with Temporary Assistance for Needy Families (TANF): How TANF Can Better Support Women’s Wellbeing and Reduce Intimate Partner Violence

**DOI:** 10.3390/ijerph19031170

**Published:** 2022-01-21

**Authors:** Rachael A. Spencer, Emily D. Lemon, Kelli A. Komro, Melvin D. Livingston, Briana Woods-Jaeger

**Affiliations:** 1 Hubert Department of Global Health, Rollins School of Public Health, Emory University, Atlanta, GA 30329, USA; 2 Department of Behavioral, Social, & Health Education Sciences, Rollins School of Public Health, Emory University, Atlanta, GA 30329, USA; emily.lemon@emory.edu (E.D.L.); kkomro@emory.edu (K.A.K.); melvin.livingston@emory.edu (M.D.L.); bwoodsjaeger@emory.edu (B.W.-J.)

**Keywords:** depression, intimate partner violence, structural discrimination, Temporary Assistance for Needy Families, welfare

## Abstract

Women experiencing poverty are more likely to face intimate partner violence (IPV), poor health, and stigma. IPV survivors are overrepresented among those who receive Temporary Assistance for Needy Families (TANF), a conditional cash program serving families experiencing poverty. More generous TANF policies may be protective against IPV, but a greater insight into TANF’s effect could be gleaned through a contemporaneous study that examines intersecting determinants of wellbeing and engages community interpretation of findings. Using an adapted Family Stress Model framework and analyzing data through an intersectional and community-based lens, we explore the impact of TANF on women’s wellbeing through in-depth, semi-structured interviews during the COVID-19 pandemic with 13 women who had TANF experience in three U.S. states. Data were analyzed using thematic analysis in MAXQDA and researchers facilitated three member-checking events to enhance validity of result interpretation. Four themes emerged: (1) Low cash and conditional benefits provided limited short-term “relief” but contributed to poverty and hard choices; (2) TANF benefit levels and conditions increased women’s dependence on others, straining relationships; (3) Women undertook extraordinary measures to access TANF, largely to fulfill their roles as mothers; and (4) TANF stigma creates psychological stress, differentially experienced by African Americans. Increasing TANF cash benefits and other cash transfers for those experiencing poverty, adopting solely state funded TANF programs, increasing funding for TANF administration, addressing TANF stigma and racialized narratives, and allowing optional child support participation or a larger “pass-through” of child support are important steps toward making TANF more protective against IPV.

## 1. Introduction

Women who experience poverty and its related stressors are more likely to experience relationships of a poorer quality, sometimes involving intimate partner violence (IPV) [1,2]. Satisfying or positive relationships can improve health [3]; conversely, IPV, or “physical violence, sexual violence, stalking and psychological aggression (including coercive tactics) by a current or former intimate partner” [4], may sustain, or deepen, individual poverty [5], due to its associations with depression, physical injury, and post-traumatic stress disorder [6,7]. Through economic abuse, abusers perpetuate survivors’ experience of poverty by, for example, interfering with employment, controlling a survivors’ resources, or obtaining debt in the survivors’ name [8].

Relative to their proportion of the general population, IPV survivors are overrepresented among participants in the conditional cash transfer program funded as a part of the block grant known as Temporary Assistance for Needy Families (TANF) [9,10,11,12]. Replacing Aid to Families with Dependent Children (AFDC) in 1996, the federal government created the TANF block grant to achieve the following aims:

Provide assistance to needy families so that children can be cared for in their own homes; Reduce the dependency of needy parents by promoting job preparation, work and marriage; Prevent and reduce the incidence of out-of-wedlock pregnancies; Encourage the formation and maintenance of two-parent families [13].

The federal government capped the federal TANF block grant at 16.5 billion dollars per year [14] and created a framework through which states would allocate TANF funds toward a conditional cash transfer program serving families experiencing poverty (colloquially known as TANF, cash assistance, public assistance, and welfare). Through the TANF block grant, states also could fund other programs, including pre-k and headstart, refundable tax credit programs, and child welfare programs as long as they met the overall TANF block grant goals [15,16,17]. Given its outsize influence on the lives of IPV survivors, this manuscript focuses on the conditional cash transfer program serving families experiencing poverty (here after called TANF), which provides recipients short-term support in the form of cash transfers, childcare vouchers, job training programs, and educational programs under multiple conditions [18].

For the TANF conditional cash transfer program, the federal government articulated a short-term, work first approach by setting a 60-month long lifetime limit on the number of months most families may receive federally-funded cash transfers [13,19,20] and by using cash incentives to encourage states to engage a percentage of TANF conditional cash assistance participants in core activities designed to prepare participants for work [21]. Additionally, legislators sought to recoup TANF conditional transfer-related expenses through child support enforcement. The TANF conditional cash transfer requires most custodial parents to participate in efforts to collect child support from non-custodial parents of children involved in TANF and assign rights to the majority of child support to the state [22,23]. Federal policymakers updated the TANF framework via in the Deficit Reduction Act of 2005 [24] but have retained the same basic structure and level of block funding for TANF since its creation.

Within the federal framework, TANF conditional cash transfer programs are managed by state and county governments, resulting in differing levels of TANF generosity and access across—and sometimes within—states [25]. In 2019, the cash benefits provided to recipients ranged from 22.7% to 81.4% of the federal poverty level [26] and states established time limits for TANF lifetime receipts that ranged from 12 to 60 months [25]. The range of sanctions, or financial penalties for those deemed to have failed to comply with work requirements for the first time, ranged from a partial reduction in cash benefits that lasted until compliance with requirements to a full reduction of benefits for the entire TANF unit for at least 3 calendar months. All states require most custodial parents to cooperate with efforts to obtain child support from non-custodial parents; however, the amount of child support received from noncustodial parents that is “passed through” to the custodial parent ranges from $0 to $200 depending on the number of children [23]. TANF access among families experiencing poverty also differs by state. In 2019, the ratio of families receiving TANF to the number of families with children experiencing poverty ranged from a high of 68 in California to a low of 4 in Tennessee and Louisiana [27]. Despite the decline in TANF access, TANF remains an important economic security policy in the lives of many. In the fiscal year 2019, 1.6 million children and 437,000 adults received TANF [28].

### 1.1. TANF Structural Discrimination and IPV Survivors

Numerous studies and policy reviews have found that a work-first, time limited, and waiver-based approach to welfare disproportionately and negatively impacts groups that are unable to meet TANF’s aims, including IPV survivors [29,30,31]. Indeed, TANF policies are characterized by structural discrimination [30], or the ways in which institutional or structural conceptions of discrimination detach behaviors from intentional agents, instead linking them to rules, procedures, and policies that differentially impact one group over another [32,33,34,35]. First, work requirements, or the mandate that participants engage in work, educational activities, job search, or training in exchange for benefits, have been found to disadvantage groups who do not have equal access to the supports necessary to participate consistently [36]. IPV survivors often face abusive interference with work participation, creating structural and social barriers to consistent engagement in employment and educational activities [9,37,38]. Secondly, enforcing policies that involuntarily remove families from TANF, such as full-family sanctions and time limits, is particularly burdensome for those who have structurally limited access to employment. IPV survivors often have reduced savings and limited employment histories owing to economic abuse [8]. Finally, requiring TANF participants to cooperate with child support petitions from a non-custodial parent creates barriers for IPV survivors who are fleeing abusive partners and fear being found by them or abused for seeking child support [39].

State and federal governments have acknowledged the potential for TANF to differentially benefit certain groups by carving out state-specific exceptions, and several states have developed solely state funded programs for groups that have barriers to employment [40]. The federal government developed one notable exception for IPV survivors through the Family Violence Option (FVO). Under the FVO, states could opt to screen individuals for IPV, refer them to services, and provide IPV survivors with exemptions from multiple eligibility criteria, requirements, and other punitive policies, such as work requirements, child support enforcement rules, and time limits [18]. The extent to which aspects of the FVO have been adopted varies across states and there is doubt about the effectiveness of the FVO in creating equitable opportunities for IPV survivors [39,41]. Further, FVO protections are generally limited in scope to three categories of exemptions—time limits, work requirements, and participation in child support petitions [41]—so IPV survivors are not exempted from the majority of TANF policies and could be experiencing obstacles that are not addressed through the FVO.

### 1.2. Intersections of TANF Receipt, IPV Experience, and Race

IPV victimization is just one of many experiences that affect the manner and ways in which participants benefit from TANF. Other studies have noted that mental health challenges, experience with homelessness, and structural discrimination associated with race, and ethnicity all impact the extent to which individuals may benefit from TANF [15,42,43,44,45,46,47,48,49]. The importance of looking at the impact of TANF using an intersectional approach, or an explicit examination of the ways in which TANF systems and policies interact with women’s multidimensional social identities, has been well articulated for decades [50,51,52]. Such a complex approach enables researchers to move beyond a single-factor explanation to explore the interplay of TANF policies and women’s complex identities.

Strict TANF policies are particularly disadvantageous for African Americans, whose higher rates of IPV [2], poverty [53], and chronic health issues, including the effects of the COVID-19 pandemic [54,55], are rooted in structural racism, or the “totality of ways in which societies foster racial discrimination through mutually reinforcing systems” [56]. The effects of strict TANF polices are exacerbated by structural discrimination in multiple systems that create barriers to TANF participation. For example, redlining into neighborhoods of concentrated neighborhood poverty [53] means that African Americans are more likely to experience housing instability, overcrowding, inadequate access to healthcare, and transportation barriers. In other words, the main reasons for noncompliance with program rules are actually structural rather than behavioral [57,58]. African Americans have faced persistently elevated levels of employment discrimination [59] and reduced access to economic supports, employment with sufficient COVID-19 protections [60], and opportunities for employment due to mass incarceration [61]. These multiple intersecting and reinforcing systems, combined with individually held and operationalized biases against African Americans, overlap and compound the toll on African American’s wellbeing and the ability to participate in, and voluntarily exit from, TANF with the employment and means necessary to thrive. Unlike the FVO for IPV survivors, no exceptions have been made to address structural racism.

Additionally, African Americans may be more likely to perceive and experience stigma, or internal and external manifestations of a devalued social identity [62], associated with poverty and TANF receipt [63,64]. Identity stigma, or “concerns about being labeled with negative stereotypes associated with recipients of means-tested programs,” ref. [63] may be more relevant for African Americans because government efforts to create TANF were imbued with and supported by the stigmatizing rhetoric of recipients being undeserving or lazy that included racialized narratives [30,65]. While African American women were at the helm of the welfare reform movement that argued for a minimum income for all Americans [66], the movement lost momentum when AFDC benefits were reduced in the 1980s and as “deserving” and “undeserving” welfare recipients were defined, often in terms of race [67,68]. Scholars note that the notion that welfare recipient is synonymous with African American race reduced support for a generous welfare system among the Caucasian, U.S. population, leading to even more restrictions over time [69,70]. The implications of stigma are numerous for economic, emotional, and social wellbeing among families. Experiencing stigma reduces access and the ability to benefit from informal and formal resources [71,72,73,74] owing to both individualized biases toward the stigmatized individual as well as structures that are not designed to meet the needs of those experiencing stigma.

### 1.3. TANF as an Intervention to Promote Wellbeing

Multiple quantitative studies have taken advantage of the differences in TANF generosity across states to examine the impact of TANF on wellbeing; however, these studies have produced mixed results. Some suggest that more generous TANF policies, or policies that provide greater access and resources, may protect against family violence [75,76] and make contributions to wellbeing among family members [77]. However, the quantitative literature is not conclusive on TANF’s role in promoting economic or emotional wellbeing [78] or violence reduction [79] and provides a limited insight into the mechanisms by which TANF policies achieve their outcomes. Furthermore, these studies suppose that TANF policies are implemented and experienced as they are written. Quantitative studies are limited in that they cannot account for individual or TANF center deviations from state-wide policies.

Overall, qualitative research, often involving women who have experienced TANF, indicates that many families are disadvantaged by TANF as a system and, in particular, by policies focused on reducing welfare participation through employment. In 1997, Edin and Lein [29] conducted a multi-city, multi-state study involving interviews with 379 single mothers earning low to very low incomes, half of whom had welfare experience. Through the framework of the family budget, Edin and Lein found that AFDC income was insufficient to meet basic family needs, causing stress and economic hardship and forcing single mothers to supplement their income through contributions from friends, family, and intimate partners, as well as poorly paid, unstable employment. Participating in employment could result in the loss of AFDC-related income and AFDC-related housing and childcare subsidies upon which women relied, potentially creating greater economic hardship than had existed prior to employment. As a result of their need for supplemental funding, women’s relationships with those upon whom they depended financially could take on a transactional quality, depleting trust, connections, and closeness between women and their family members and intimate partners. Concluding that, “as a safety net, TANF failed these women,” (pg. 10) Seefeldt’s [47] 2013 interviews with single mothers in Michigan revealed multiple ways in which TANF did not support women to achieve their economic or employment goals. In particular, TANF’s rigid employment policies, failure to provide adequate support to women facing mental and physical health challenges impeding employment and TANF participation, and implementation of time limits regardless of women’s financial circumstances “did not help [women] find jobs…did not assist with personal and family challenges…and failed to perform as a safety net.” Focusing on IPV survivors’ experiences with TANF in Georgia, An and Choi’s [39] interviews with survivors, advocates, and IPV experts echoed prior research that the TANF waiver-based system rooted in the FVO is poorly designed to address individual needs related to participation in TANF and employment [31,80]. Few women in the study were screened for IPV as part of the TANF application process and so were not considered for waivers from work requirements or the emotional and instrumental support that would help them safely access TANF. When women revealed their IPV experience, often they were not provided with individual waivers and felt coerced by caseworkers to decide whether to pursue their TANF case without full knowledge of their likelihood of receiving waivers under the FVO.

While the literature on TANF and wellbeing is rich, the timing, methods, and scope of extant studies suggest that the relationship between TANF and women’s wellbeing deserves continued attention. First, many studies were conducted when TANF was first adopted—approximately 25 years ago—when TANF offered higher levels of cash benefits and more families accessed the program [27]. The decline in TANF access and generosity, combined with the unique hardships associated with the COVID-19 pandemic, suggests an updated, qualitative study could provide additional insights. Second, researchers are often the main or only arbiters of study results. Exploring the credibility and validity of results with community stakeholders who have lived experiences with TANF could offer unique insight into quotes, themes, and results [81]. Finally, most studies are based on empirical evidence rather than sound, theoretical models that could provide guidance as to the mechanisms by which TANF influences wellbeing.

This community-based study seeks to begin to address gaps in the TANF-wellbeing literature using semi-structured interviews with women to explore the relationships between TANF and women’s wellbeing and intimate partner relationships and how these relationships are moderated by structural discrimination. We use the Family Stress Model (FSM) [82,83] as the guiding framework for the study because it provides important insights into the linkages between experiencing low income, wellbeing, and family dynamics, including IPV [84] and TANF, which have been shown to relate to multiple constructs contained within the FSM [20,75,85]. Specifically, the FSM creators hypothesize that *negative financial events*, or “acute financial stress created by unfavorable changes in economic circumstances” (e.g., being sanctioned or experiencing a time limit that reduces family income), combined with *low family per capita income*, will produce *economic pressure*, or objective measures of economic hardship (e.g., inability to buy necessities or eviction due to lack of payment) [83]. Economic pressure contributes to *psychological distress*, defined as poor mental health “ranging from normal feelings of vulnerability, sadness, and fears to problems that can become disabling, such as depression” [86] and *caregiver relationship conflict*, defined as “behaviors that reflect both aggressive and angry responses, such as criticism, defensiveness, and insensitivity and the withdrawal of support” [83]. The FSM is well-tested, with studies finding that the model could be fitted to the experiences of recipients of multiple racial and ethnic groups [37,86]. Guided by the FSM, our study seeks to answer the following questions:

(1) How do women perceive that TANF policies influence women’s wellbeing (economic pressure and psychological wellbeing) and their relationships with intimate partners?
(2) How are the relationships between TANF policies and women’s wellbeing and relationships with intimate partners influenced/impacted by structural discrimination?

## 2. Materials and Methods

### 2.1. Study Setting

Participants in this study primarily had experience with TANF in three U.S. states—New York, Missouri, and Kansas. While we do not seek to make explicit comparisons between women’s experiences across states, we sought out participants in diverse TANF policy environments in order to capture experiences of different TANF policies as they relate to our theoretical model (Table 1).

### 2.2. Community Engagement

In developing, implementing, and analyzing data for this community-engaged study, the research team worked with two community-based organizations located in large cities. One organization is located in the northeast and provides services to individuals who have experience with IPV or gender-based violence. Another is located in the mid-west and is an early childhood education head start that provides services to families, many of whom have experience with IPV. The community partners were involved in reviewing and commenting on the protocol, supporting the development of the interview guide, and referring participants. A total of three member-checking events with professionals at the community-based organizations that partnered with the study team and women who also have experience with TANF as recipients were conducted to increase confidence in validity of the interpretation of study results [81]. During member-checking events, the Principal Investigator (PI) sought to confirm results as interpreted by the research team by sharing synthesized, analyzed data from the interviews, seeking agreement but also opposing opinions, and providing opportunity to add data to the analysis [87]. Events took place over Zoom because of travel restrictions related to the COVID-19 pandemic. The PI reviewed the purpose of the study, read the synthesized themes along with de-identified, illustrative quotes, and then asked participants to respond to the following questions: What do you understand this quote to mean? To what extent and in what ways does this quote reflect the overarching theme? How does this theme reflect your lived understanding of women’s experiences with TANF? Participants provided their feedback and, since both previous TANF recipients and service providers could speak from their own personal experiences, participant feedback was given equal weight in analysis regardless of its source. Notes were taken by two members of the research team and then the study themes were consolidated and revised after each event.

### 2.3. Participants 

The participants in this study are 13 mothers who received TANF for themselves and/or their child within the recent past, defined as having received TANF in the past 7 years. Both purposive and snowball sampling procedures were used because we sought to recruit individuals who have experiences that are relevant to our research questions [88]. Snowball sampling is especially useful when populations of interest are hidden or difficult to recruit because they are involved in sensitive activities [89]. With regard to this study, snowball sampling was used to address recruitment challenges associated with the dwindling size of the population with TANF experience [17], social stigma associated with TANF receipt [63], and formal institutional barriers to accessing participants due to social distancing protocols associated with COVID-19. Primarily, participants for this study were referred by two community-based organizations (CBOs) that partnered with us in the development of the protocol. CBOs were asked to refer women to the study if they had “recent” TANF experience—an amount of time that could be defined broadly by CBOs—regardless of their relationship or IPV history. TANF experience became the primary qualifying factor for women referred to the study because so few adults receive TANF—in 2019, nationwide only 23 out of 100 families in poverty experienced TANF and half of these families receive TANF only for children. Women could also be referred to the study via snowball sampling, operationalized by the PI as asking women who completed the interview to refer other eligible women to the study. Women referred to the study were then vetted for eligibility by the PI before being recruited.

### 2.4. Field Methods

In-depth semi-structured interviews were selected for the current research because we sought to understand individual experiences with, and perceptions of, TANF policies [90,91]. Due to restrictions associated with COVID-19, recruitment and interviews were conducted virtually over Zoom, based on the technology available to the participant. Research indicates that phone interviews yield quality data on par with data gathered from face-to-face interviews [92,93]. When interviewed, the participant was located in a private location of her choice. The PI who conducted the interviews was located in a private office.

The personal and sensitive nature of this study required thorough ethical considerations to ensure that the research was not coercive or did not place participants in danger. By the nature of recruiting women directly through service organizations, women were already connected to a system of supports. With the participants’ permission, the PI was prepared to connect the participants with resources in coordination with the community-based organization to manage distress that may occur during or because of the interview. No participants requested to be connected to resources as a result of stress and, unprompted, most indicated that they appreciated the opportunity to speak about their experiences. Furthermore, due to the precautions associated with the COVID-19 pandemic, all interviews were conducted remotely over Zoom when the participant could be in a safe, private location [94]. The Emory University Institutional Review Board approved the research.

Informed consent was obtained and documented by the PI, a doctoral candidate, who read the informed consent document to the participants, provided time to answer participants’ questions, and asked a series of questions to ensure that informed consent could be given. For example, participants were asked to describe the purpose of the study, what they would be asked to do if they joined, and to practice how they would respond if they did not want to answer a question or stop the interview. The PI then documented consent in an online, HIPAA-compliant platform. Interviews were audio recorded with the verbal permission of the participant. Each participant received a $50 gift card as remuneration for her time and contributions to the study.

The interview began with a calendar landmarking exercise to help improve the recall of events involving TANF applications, participation, and experience of TANF policies. Calendar landmarking exercises, also referred to as life history calendars [95], are used widely in the social sciences [96] to improve participant recall of retrospective events compared to other techniques [97], even when the period of inquiry is within the past two years [98]. This approach serves to support recall by providing *bounding cues*, or temporal points, against which the domains of interest can be anchored and *sequencing* events [99], which is the process of identifying what happened before or after an event to “reduce the risk of omitting events,” and enable the establishment of linkages between domains of research inquiry via *top-down* and *parallel retrieval* [96]. The calendar-landmarking exercise has been used to improve the recall of IPV events among IPV survivors and is particularly appropriate when inquiring about events that have occurred throughout the life course [100].

Subsequently, the PI conducted a standard, semi-structured interview using an interview guide. Specific questions were asked of the participant based on her responses to the calendar landmarking exercise and more general questions were asked of all participants. Finally, participants completed a brief demographic form covering information about their age, race, ethnicity, experiences with other forms of governmental assistance, and health.

### 2.5. Measures

The interview guide was developed by the PI based on the constructs in the FSM and was revised and edited based on feedback from the research team and three different community partners who have experience providing services to IPV survivors. The guide covered multiple domains, including participants’ experience with TANF, effects of TANF on intimate relationships and wellbeing, and perceptions of TANF policies (Table 2). The study focused on several primary TANF policies of interest—cash benefits, time limits, sanctions, and mandatory child support participation—because these policies have been posited to relate to IPV and women’s wellbeing. Using open-ended questions, we also encouraged participants to identify additional policies that may have affected their TANF experiences. The interview questions were worded to explore the FSM framework and assumed that events described by the participant affected mood, relationship wellbeing, and/or economic wellbeing. At times, when prompted to describe the stress, participants noted that events related to TANF did not affect their wellbeing. Responses that the events had no impact on these three constructs were noted and the PI inquired as to the reasons behind the effect or lack of effect in subsequent questions.

### 2.6. Data Analysis

The interviews were anonymized and transcribed verbatim by the PI. Data analysis was conducted using thematic analysis [101] to identify and explain descriptive themes across interviews relating to the experience of TANF policies to women’s wellbeing and relationship quality. We also characterized women by their self-identified race, experiences of IPV, and state of TANF experience to look for themes that emerged across these diverse characteristics and for indications of areas to explore in future comparison research with a larger sample. Structural discrimination was considered present if a participant described how an obstacle to accessing TANF benefits was associated with a stigmatized identity (e.g., being an IPV survivor).

The PI-created codes, or analytical labels to related instances of data, to create structure from unstructured data [102] in order to identify emergent themes and explore the application of the current study’s theoretical model. The PI developed a codebook by first creating analytical memos and noting common themes that emerged from the data or that mapped onto the theoretical framework, then creating and defining codes from a subset of three transcripts [91], one from each state represented in the data. The codes were primarily deductive in nature, drawn from the FSM (e.g., psychological wellbeing) although inductive codes (e.g., instrumental support) were also identified. Finally, the PI organized a total of 31 codes into a coding tree using MAXQDA [103], a qualitative software analysis tool.

The PI then coded three transcripts that were not used to create the codebook and ensured that the transcripts represented women who had experience across different states—New York, Kansas, and Missouri. A second coder, a doctoral candidate at the same university and a member of the research team, recoded two transcripts using the codebook and the PI-calculated interrater reliability using Cohen’s Kappa in MAXQDA [104]. Because the initial Cohen’s Kappa did not indicate a high level of agreement (≥0.8) [105,106], the PI and second coder met to understand where differences existed and explored underlying causes of the disagreement (e.g., ambiguity in the codebook definitions, different perspectives and experiences of the coders) [107]. Once consensus was reached, the PI updated the codebook and then both coders coded two additional transcripts to calculate the interrater reliability and met again to discuss differences. Updates to the codebook were made and the process was repeated one additional time until the *a priori* Cohen’s Kappa statistic (≥0.80) was reached. The PI and second coder then independently recoded all of the transcripts, calculating Cohen’s Kappa after each transcript was double coded and disagreements in coding were reconciled. The PI selected the final coded transcript used for analysis. For all 13 interviews, Cohen’s Kappa ranged from 0.81 to 0.95.

Codes were added and redefined as needed using constant comparison until thematic saturation was reached. For this study, thematic saturation was conceptualized as the point at which “no additional data are being found whereby the (researcher) can develop properties of the category” [108]. Our focal categories included the key TANF-related issues and experiences as defined by deductive codes in the FSM. While we used data from participants across diverse environments to achieve this level of saturation, we were not seeking saturation in terms of how these issues differ by policy context or even within a specific policy context, which would have required a much larger sample size. We operationalized thematic saturation for our categories of interest as the point “in data collection and analysis when new information produces little or no change to the codebook” [109]. There is no universal, minimum number of participants necessary to achieve thematic saturation in a non-probabilistic qualitative sample. However, studies have demonstrated that as few as 12 in-depth interviews are needed to reach thematic saturation as we have operationalized it, especially for an investigation of higher-level concepts [109,110], as is the case with this study. Similar to Guest and colleagues [109], we tracked changes made to the codebook during the double coding process to identify the point at which no new codes were added or refined. No new codes were added after the seventh transcript when the two coders revised the codebook for a third time, and no codes were refined after the tenth transcript was analyzed by both coders. Three additional interviews were completed to ensure that no new codes were required or refined. The analysis of the three additional interviews confirmed that the codebook was stable and interview concepts could be adequately categorized using existing codes (i.e., no additional codes were needed or refined). Therefore, we concluded that thematic saturation, as defined in this study, had been reached. The calendar landmarking exercise was recorded for reference only.

Member-checking with professionals at the community-based organizations that partnered with the study team and women who have experience with TANF as recipients then took place to increase confidence in validity of the interpretation of study results [81]. The PI presented the main themes and supporting quotes and members gave their perceptions of the interpretation and added context to the themes and quotes. Member-checking occurred three times: once with 10 advocates and case managers at the site in the northeast, a second time with 18 case managers, therapists, and advocates at the site in the Midwest, and a third time with 6 women who have experience with TANF, but did not participate in the interviews. While no changes to coding were needed, the member-checking phase provided additional context to the study results and suggested that multiple sub-themes were representative of higher-order constructs and thus could be synthesized into main themes. After the three member-checking events occurred, the research team further synthesized the data, reorganizing the results under 4 main themes.

## 3. Results

On average, the thirteen participants were 33 years old and had primary experiences receiving TANF for themselves and/or their child in New York (n = 5), Missouri (n = 6) or Kansas (n = 2; Table 3). Sixty-nine percent of women (n = 9) had open/current TANF cases and, among the four women who had a closed TANF case, their last period of TANF receipt ended, on average, 3 years prior (range: 1 year to 7 years). Some women had received TANF in multiple other states, including Florida and Colorado. With the exception of one participant with primary TANF experience in Kansas, all women were recruited by our community partners. The one individual recruited via snowball sampling was similar to most other participants recruited by community partners in that she had similar demographic characteristics (age, race, number of children), family structure, and TANF experience within the past year. In total, the landmarking and interview questions lasted an average of 61 min with a range between 40–80 min.

**Theme** **1.** *Low cash and conditional benefits provided limited, short-term “relief”, but women continued to experience poverty and hard choices*.

All women reported experiencing short-term positive emotions and improved psychological wellbeing when cash benefits were deposited into their accounts. Seven of the 13 women used the word “relief” to describe how they felt when they confirmed deposits of the cash benefits. Receipt of the monies enabled them to pay basic bills (e.g., telephone) and buy food for their family members. Most of the monies were spent on taking care of the children’s basic needs. The TANF monies were spent very quickly to tend to basic needs with little, if anything, left over for savings.

I feel good. I feel like, yeah, thank God. I feel relief!… I’m like yeah. It finally came. It means I can get this. I can get that. I can use this to get that with it now. You know. Almost like your paycheck. But it comes once a month on a card. And you be happy when you get it and then it be gone right afterwards, but hey.—African American/Black Participant, Missouri

TANF cash resources were frequently described as “enough to cover just the basics. If you have outside help though” (African American/Black Participant, Missouri). Seven women discussed negative emotions associated with the low sums of money they received and the resulting residual economic stress that they experienced. Two described feeling stress because they had limited governmental and social support and therefore, they had high levels of residual economic pressure after spending TANF monies. One felt angry because she had paid into the system as a worker and she received so little from TANF in return. Another woman described feeling depressed because she was “broke” (African American/Black Participant, New York) and the only person in her family receiving TANF.

I would immediately run down a list in my brain of what we were low on and what we needed. And then I kind of got a sense of relief of some of that stress being gone because I was gonna have some money coming in. But then also feeling like, what is this? Cause when you’re not working and that’s really the only income that you’re getting, it’s kind of also at the same time stressful, because you only have so much but you need so much.—Mixed Race, African American/Black and White/Caucasian Participant, Missouri

Due to their needs exceeding their individual resources, women were often forced to make hard choices and tradeoffs, including choosing between their current and future wellbeing. This resulted in some women voluntarily separating from TANF to bank months for future use in the case that they “really” need it. Banking months meant that women felt increased economic pressure at the loss of TANF income in the short-term, but increased their options for the future.

I think I only have four more months [of TANF] left. So I decided to go and get myself taken off [TANF]… because otherwise when the time comes that I really need it, I ain’t gonna be able to have it right there. Because say like, right now… I could uh, work, go back to work, I can go to school in the evenings part time. I can look into maybe. I really don’t know, like what I’m going to do with the kids as far as school yet. But I’m still trying to figure it out. Really don’t even know if it’s the best time to really get taken off, but to me, I know it’s either now or never… I don’t want to use it all up and then… I get pregnant and I need it again. And I can’t work.—African American/Black Participant, Missouri

Low levels of cash benefits had multiple implications for women’s intimate relationships. Limited benefits forced three women to contact abusive partners and seek resources, resulting in additional abuse. Even though cash benefits were not sufficient to cover all of their expenses, most women indicated that they were not deterred from leaving their violent relationships because their decision to leave was rooted in fears for their safety, the occurrence of a major violent event, or impact on their child’s wellbeing. However, one woman felt the instability and residual pressure associated with low amounts of TANF cash benefits made her regret leaving her in a more economically stable but violent relationship.

I have had moments, to be honest, and I don’t have them anymore, but I’ve had moments in the past when I was like, shit I just should have stayed. I should have shut my mouth put up with it and should have stayed cause at least he was paying the rent. That’s how that made me feel. To actually look back at someone that was violent, chaos, and instability in every way, sense and form and to think I should have just stayed because at least I was secure.—Mixed Race, American Indian/Latina Participant, New York

Three women described feeling negatively about TANF because they believed that the limited cash benefits provided by TANF perpetuated cycles of poverty. In particular, they described how the combination of limited cash benefits, significant work requirements, and the overall burden associated with TANF (e.g., paperwork, mandatory meetings) ensures that those experiencing poverty will remain in poverty.

With programs like that, you know, they want you to do the bare minimum and it’s kinda built to hold you in poverty or to keep you at your lowest. And it was kinda like finding out, you know, applying for certain programs or being in certain programs and learning that, you know, that’s the whole point of programs like that.—African American/Black Participant, Missouri

**Theme** **2.** *TANF Low Benefit Levels and Conditions Increased Women’s Dependence on Others, Often Taxing Intimate Relationships.*

Women valued TANF income because they could use the cash and resources to achieve some level of independence and contribute to the household; however, low levels of benefits and TANF conditions meant that women still had to rely on social and instrumental support from family members, friends, and community organizations. For example, TANF work requirements that were distant from women’s homes or were conducted outside of regular daycare hours often made women reliant on others to assist with childcare, transportation, and meal preparation. Women’s reliance on support from family and friends to address resource gaps while receiving TANF sometimes created conflict in, or added stress to, women’s intimate relationships. Women felt stress when they had to ask others for help—especially for help with their child—and also knew that the support on which they relied could be withdrawn, resulting in them taking extra care to maintain their relationships.

It was difficult, you know, [redacted] is my baby. So it’s just kind of hard to like, I mean, my parents, they’re fine with it, like, you know, her daycare was literally right around the corner. So it wasn’t like, you know, it was like something out of the way for them. But then it kind of got out of the way, cause you know, they would have to get her dressed, take her there. It was just a lot. So it was like it was kind of stressful that I couldn’t take her to school myself and, you know, just go to work after. It was stressful.—African American/Black Participant, New York

Women experienced conflicts and psychological stress when TANF required them to participate in the initiation of child support petitions from the non-custodial parent of their child. Seven of the 13 women experienced this requirement. While women had the option to indicate that they did not know who the non-custodial father was, only one described taking this route because she was afraid of the father of her child. Others revealed the name of their child’s father because they supported the idea of the father of their child contributing via child support, wanted to comply with the TANF system, or feared losing TANF benefits for perceived lack of compliance.

So once they give you the public assistance they automatically tried to put him [my child’s father] on child support… Um, basically they told me, like, if you want to keep getting public assistance, you have to give us the name or give us some sort of information so we can contact them. Or you can simply say you don’t know or you just don’t know who your child’s father is. But if they feel like they know who your child’s father is and you’re not giving them information like basically they threaten to cut you off of the public assistance.—African American/Black Participant, New York

Women whose TANF receipt resulted in the initiation of a child support petition mentioned receiving confrontational calls from the non-custodial father of their children. Women described the calls they received as “horrible” (African American/Black Participant, New York), in part because of the abuse they received for the initiation of the petition and also because they often had to explain the TANF system and its child support requirements to the father.

In fact, the first time I ever had an issue with child support, actually coming and doing their job was the month before I was getting off of it and going into work with my new job. My daughter’s dad called me cussing and yelling… And he’s like, ‘Man, you got to talk to these people.’ I’m like, ‘What are you talking about,’ ‘They [TANF] just took my money.’ So we had a phone conference and they were trying to get help for child support for my TANF that I had been on for the past three years, but I was getting off of it. I had used up my lifetime, and I found a job that paid enough… And um yeah I basically had the vouch for him.—African American/Black Participant, Missouri

Many women felt that initiating child support petitions while receiving TANF placed unnecessary stress on family relationships, made co-parenting significantly more difficult, and decreased the amount of monies and resources they would receive in total to care for their children. One woman felt that initiating a child support petition caused a chain of events that increased the family’s interactions with the court system and reduced the money that her child received from the non-custodial father.

With the child support, you know, they [non-custodial dads] miss a payment that becomes, you know, they can get jail time or they lose things. And if they don’t pay it, then not only this child support not get paid, but I don’t receive anything either.—African American/Black Participant, Missouri

For IPV survivors, just the existence of the child support requirement and knowing that they might be asked to assist in a child support petition created psychological stress and structural barriers to TANF receipt.

When the [COVID-19] pandemic first really hit hard, I was like, I’m thinking about applying to TANF… but I’m hesitant because I don’t want it to trigger any type of child support modification against my daughter’s dad, which in some cases, depending on the amount of TANF versus the child support, it can trigger a child support modification… Anytime they do anything… like the fact that now they’re taking 140 a month, rather than 117 a month, there tends to be some backlash.—Mixed Race, Caucasian/White and Pacific Islander Participant, Missouri

Women could be exempted from the child support requirement if they lived with the father of the child or if they asked for an exemption because of their experience of IPV. Two IPV survivors sought and were granted exemptions from the child support requirement; however, the process was psychologically distressing. One survivor described feeling as though the case worker did not believe her and the second described feelings of “terror” waiting at least two weeks before she received a disposition on her request.

I was terrified. I mean I had like cried for like three days before this because I’m like oh my God this is going to give him rights and he’s gonna hurt my kid like I was just… You know, I was losing it. And um, when I went in, I explained to them, I was like, look, I’m this is the situation. And I was like, he’s already beat one of his girlfriends and she ended up losing her kid… They ended up giving me this paper and it said we are we are denying, or we’re closing the case due to harm to the mother of the child. So they ended up closing it and I haven’t heard anything about it.—Caucasian/White Participant, Kansas

For some participants, low levels of TANF benefits and limited child support passthroughs caused some women to ask the father of the child to provide child support directly. Especially if the father was already mandated to pay child support to the state, such requests could create moments of abuse and conflict.

Because he [the father of my child] is still having to come out of pocket when I need stuff for [my child] and I can’t do it… It makes, it always gets thrown back in my face. It causes a lot of arguments to an already tense relationship… He’ll be like, I don’t understand why I have to give you money and the state money. He’ll mention about how, he’s even said in the midst of like a heated argument, how he regrets having a child in the first place because of all of this.—Mixed Race, African American/Black and Caucasian/White Participant, Missouri

TANF caseworkers could be a source of support for women; offering both instrumental and social support in times of difficulty, including experiences of IPV. However, women’s relationships with caseworkers differed tremendously across individuals and states of TANF experience. None of the women in New York had a consistent caseworker with whom they had contact, or even knew they had been assigned to a certain caseworker. These women described their experiences interacting with TANF as inconsistent, frustrating, and largely unhelpful because they had to share their experiences with a new person each time they went to the office.

You are not assigned to the same person [caseworker] all the time. So you get, you get a different person when you go back… I would like someone who, she or he has seen me for years and they know me… But you know, not knowing these people, I have, I have no choice, but to not share myself with them.—African American/Black, New York

In contrast, the women in Kansas and Missouri who reported ongoing, regular contact with the same caseworker were more likely to describe having positive, productive relationships with their caseworker.

[My caseworker is] like my mom in the system. So it just, I felt comfortable enough [to tell her about my experiences of IPV] and I knew that if I opened up to her about what was going on, she would make sure that I got the resources and stuff that I needed and was in contact with the right people… every time we would meet or talk, just how much genuine care and love she would show for me and my kids… I had went through a situation where I didn’t have daycare for my child and I wanted to go to a job interview and she allowed me to bring my kids to her office long enough for me to go to my job interview. So she’s just gone above and beyond her role for me and it’s just been a lot. It’s been great because I don’t have family here.—Mixed Race, African American/Black and Caucasian/White Participant, Missouri

Even in situations where women knew their TANF caseworker and had regular contact with the same person, women’s dependence upon the actions of that caseworker could create a stressful relationship. The discretion that caseworkers can exhibit in terms of sanctions, work requirements, decisions around time limit extensions, and methods of conflict resolution created a significant power differential between caseworkers and recipients.

I felt like I was going to be kicked off of TANF because I, me and her [my caseworker] had got in the fight. So yeah, I thought that I was going to lose my TANF um but turns out I didn’t… I, I just was like, well, you can’t fight with the person that’s giving [TANF] to you and you know and then still receive it. But I if I’m not mistaken. I told her that I was worried about that. And she told me that if things between me and her wouldn’t have gotten better… I would have just switched you to another worker.—Caucasian/White Participant, Kansas

For women who lost access to TANF owing to sanctions or time limits, their connectedness to formal supports, like subsidized childcare, Food Stamps, Housing Choice Voucher Program (formerly known as Section 8 Housing), and Disability, moderated the negative impact of women’s experiences of involuntary separation from TANF to an extent. Only those who experienced TANF in Kansas and Missouri reported experiencing involuntarily losing access to TANF due to time limits. Three women had their cases closed due to their experience of TANF time limits and an additional two had less than one month of benefits at the time of the interview before reaching their time limits.

My benefits ran out in June, I believe. So my food stamps I would take half of them, fill up my house, and because I wasn’t at home all day, thanks to the daycare providing lunch and then the parent groups provided breakfast, I would eat there. And my kids are not at home. I was like, half my food stamps and I would do catering and sell dinners and that would be my extra income. And I did that straight for three months around schooling and around parent groups.—African American/Black Participant, Missouri

Three women who were employed, but had limited social support, indicated that they felt psychological stress owing to the loss of access to the safety net due to time limits. Income and asset limits associated with TANF, Housing Choice Voucher Program (formerly known as Section 8 Housing), and Food Stamps restricted women’s ability to save for unexpected expenses and so they continued to live paycheck-to-paycheck.

Luckily, I have Section Eight. So when [I stopped receiving TANF checks], my rent went to zero dollars. And then I was still able to receive my food stamps, though, and that’s scary too like if I don’t have Section Eight, sometimes I don’t know what I would do… But the thing, even with that is like when you start working, they, they take, take, take, and don’t let you get back ahead of things.—Caucasian/White Participant, Kansas

Two women reported being unaffected both emotionally and economically by the existence of time limits because they had access to a combination of employment opportunities and family support. They believed that they could make up the lost TANF income through increased employment hours and had family to assist with childcare and other needs while they increased their workloads.

[Reaching time limits] just went from me like working maybe like two or three hours in the morning to me working an actual day and getting off at normal times. I will still able to get off in time for daycare. Even if I didn’t… I could still, you know, had people to call on and to assist me.—African American/Black Participant, Missouri

For women experiencing ongoing IPV and homelessness, psychological and economic stress was particularly high due to their limited social support and formal support networks. The stress of homelessness was compounded by TANF rules that required women to provide a physical home address in order to obtain access to TANF. Several women who experienced homelessness related to IPV therefore lost access to TANF during this difficult transition.

And then I was going through, I had ended up leaving him from a domestic [violence incident] and so, I… was living in a low income based apartment and I ended up losing that because of it [domestic violence]. In the midst of that they had also cut my TANF off, not because I wasn’t working, but because I had um. I didn’t turn in an annual review on time. When I had told them that I was bouncing from place to place and trying to get myself together, they wouldn’t renew my TANF until I had a valid mailing address… It was very overwhelming. Especially with me, like finally being able to flee a couple weeks later from that domestic and not having nothing.…cause if I could’ve used that money at any time, that probably would have been the most time that I needed it.—Mixed Race, African American/Black & Caucasian/White Participant, Missouri

**Theme** **3.** *Women went to extraordinary measures to access TANF, often to fulfill their roles as mothers.*

All women discussed applying for TANF because of their need to pay for expenses related to caring for their children. One woman discussed feeling that the TANF monies were there to pay for the custodial parent’s portion of the child’s necessities, regardless of the contributions from the noncustodial parent.

Some people just aren’t with the father. That doesn’t mean the father doesn’t take care of their, you know, their portion of the responsibility. But you as a mother has to go on public assistance so you could take care of your part of the responsibility.—African American/Black Participant, New York

When asked how they budgeted their TANF benefits, women described spending most of their TANF resources to pay for their children’s needs, disregarding their own needs for long stretches of time. For some women, letting others know that they spent TANF money on their children’s needs rather than their own could be a protective strategy. Two women indicated that their abusers did not seek control of their TANF benefits because women made sure that their abusive partners knew that their benefits were small in amount, were being spent on children, and did not benefit the mother directly. Even when women’s partners demanded TANF monies as part of their controlling and abusive behaviors, women still managed to save enough money to care for their children.

Um, I think when I first was with [my father’s child] and it happened, he was on drugs pretty bad. So sometimes he would, if that money hit, he would be like it’d be 12 in the morning and he want me go to the thing and get some cash out. And that’s another reason why, like I had to leave him…. I mean I was always I was always glad that [TANF money] was coming in because that’s what I had, you know, I could pay my rent and take care of my bills. But I can say that I didn’t have to worry about like not doing that stuff. I didn’t let him take it to where I couldn’t pay my stuff. I always budgeted out like what I needed first. So I always made sure that me and my daughter was taken care of through all that.—Caucasian/White Participant, Kansas

Women described making multiple efforts to comply with TANF and maintain access to benefits explicitly for the purpose of caring for their families. Of the thirteen women, eleven women experienced work requirements, such as job searches, job training programs, and educational classes, that could result in a work-related sanction. Their fear of the psychological stress and economic pressure associated with sanctions drove women to overcome high barriers to participation. Women described walking long distances to attend appointments, relying on others in their family to care for children while attending work to meet TANF requirements, and bringing their infant child with them while working.

So there is a year where I didn’t have daycare for [my child] and he couldn’t get in until he was damn near one. So I had to… push him around the stroller while I did my volunteering… I was kind of irritated with it because I’m like, Wow, I can’t believe I have to do this with my son like and I got to clean and he’s right here.—Caucasian/White Participant, Kansas

While motherhood was a driving force for women’s TANF receipt, TANF program requirements related to work and the short-term nature TANF receipt were poorly suited to support mothers. For many women who lacked childcare and friends and family who could offer support, experiencing school and daycare closures during COVID-19 meant that they had no choice but to stay home without work and experience the pressures of deepened poverty.

I haven’t paid my rent in a while. But I’m still waiting… I would like to say that it hasn’t affected me at all, but I would be lying. I know that it’s not true. It has affected me mentally, physically, I just I’m a go getter. You know, I work hard because I believe in an honest day’s pay. I just now, not being able to send my son to school and not being able to work and COVID all these things. It has mental toll on me and you know, my body.—African American/Black Participant, New York

**Theme** **4.** *Stigma Associated with TANF Receipt Creates Psychological Stress that is Disproportionately Experienced By African American Families.*

The act of applying for and receiving TANF was associated with psychological stress for all women. Across states, the majority of women described experiencing shame or embarrassment during the application process, while using the benefits, and times they went to the state offices to handle case-related issues. They and others around them discussed the stigma associated with benefits receipt.

I mean, for me, it was just embarrassing. I mean, this whole situation is, um, embarrassing. I was working before like right before I got pregnant. During my pregnancy. I was working and it’s just like I couldn’t work because I had a lot of issues in my pregnancy. So, after a while, I couldn’t work. So that’s so, he [the father of my child] wasn’t helping me. I had no choice but to turn to public assistance.—African American/Black Participant, New York

Women’s circle of supports expressed a wide range of reactions to women’s receipt of TANF including indifference, encouragement, and strong, negative feelings about women’s receipt of TANF. Two women indicated that their partners described women’s receipt of TANF as inappropriate or somehow shameful.

His [my boyfriend’s] preconceived notion was always that people who remained on this [TANF] were either abusing it or lazy and never want it better for themselves.—African American/Black Participant, Missouri

For three participants, the stigma of participating in TANF was tied closely to the racialized narratives focused on African American and Black TANF recipients. One participant who did not identify as African American or Black felt that her caseworker did not believe her and conducted several home visits “because of the area we used to live in that area was predominantly low income black people… to where she [my caseworker] thought, okay, this is a young person trying to play me over” (Mixed Race, Caucasian/White and Pacific Islander Participant, Missouri). Women who identified as Black or African American described bearing the brunt not only of TANF stigma, but also perceptions that they were undeserving of TANF or dishonest, working the system because of their race.

That was one of the first time it was so evident that, despite you [TANF caseworker] working in this field, you don’t really believe in what you’re doing. And that this case worker might be experiencing some burnout from all the people who use it for whatever fraudulent reason. Which being African American, you know, the stigmas are tied to that. African American people really aren’t welfare queens.—African American/Black Participant, Missouri

In New York, one IPV survivor could only qualify for a housing voucher designated for IPV survivors if she obtained and maintained an active TANF case. For her, being forced to obtain a TANF case reinforced the degrading stigmas associated with poverty, racialized narratives, and IPV experience.

Because we low, low income, like we modern day, this is like, this like modern day slavery. It’s it’s not like enslaved, but it’s like yeah cuz it’s like you forcing us, you you constrict us like again. Why do I have to be I’m running for money? There’s police reports, there’s court dates. It’s all that. It’s the legal. It’s a big thing. Why do I have, then to turn around and admit to the government, hey, hey, hey. You done whooped my ass and then took everything. Like I need the [housing] voucher, just so, you know, I could provide something different for us on that case, you know, I don’t have to keep putting [my child] in that environment. Because according to the government, if I keep putting her in that environment, you know, that is neglectful and that is a dangerous and that is a whole ACS [Administration for Children’s Services] case.—African American/Black Participant, New York

## 4. Discussion

On the whole, women indicated that TANF cash benefits provided immediate but short-term relief from psychological stress and economic pressure, but that poverty, hard choices, and challenging interpersonal relationships persisted because of low levels of cash assistance, stigma, and conditions associated with TANF. In this study of participants with TANF experience across different states, findings were consistent and point to multiple ways in which TANF creates structural barriers to women’s safety and ability to thrive based on women’s multiple social identities, including race, relationship status, and IPV experience. Women who lacked access to formal and informal supports, an experience made more likely by IPV experience and structural racism, reported particularly high levels of psychological stress, conflict, and economic pressure. Despite the racialized stigmas associated with receipt and the increased levels of conflict with intimate partners, women exerted a significant effort to receive TANF, a program ill designed to support caregivers and IPV survivors. Increasing the amount of cash transfers to individuals experiencing poverty, creating solely state funded TANF programs to expand access to those who are experiencing barriers to employment, actively seeking to reduce stigma and racialized narratives associated with TANF receipt, and allowing women to opt-in to participation in TANF child support requirements and/or receive greater child support pass-throughs are important first steps toward making TANF more beneficial and accessible to all participants, but especially stigmatized groups including IPV survivors.

Overall, our study reveals the potential of cash transfers to reduce economic pressure, psychological stress, and IPV. Among a diverse group of women, we found that women’s struggles were frequently rooted in their experience of poverty. TANF cash benefits are not enough to raise a family above the poverty level [26] and, consequently, women in this study continued to experience economic stress, which has been linked to poor mental and physical health [111]. Increasing cash transfers to families has been associated with multiple benefits including increased family wellbeing [75] and reduced IPV among TANF [112] and non-TANF participants [113,114]. There are several mechanisms by which cash transfers to those experiencing poverty can be increased both in terms of the amounts provided and access to transfers. At the state level, legislators could implement refundable Earned Income Tax Credits [79] and refundable Child Tax Credits [115] that provide monthly payments for families earning wages and have been associated with reduced violence and child poverty [78,79]. State lawmakers can also make several changes to the TANF system to allow families receiving TANF to earn more and keep more of what they earn. For example, state lawmakers could reallocate TANF resources away from social service programs funded within the larger TANF block grant to increase the maximum allowable cash benefits paid to TANF recipients. TANF could also be designed to disregard participants’ tax credits (e.g., EITC), child support, and earned income, which are sometimes counted as income or assets during benefit calculation [17]. Studies of cash transfers caution against offering payments that are considered large relative to the average income of surrounding households only to one group of individuals (namely women); such payments could potentially increase IPV and conflict over how to spend income [113,116]. Multiple efforts could be undertaken to address the potential risk associated with TANF benefit increases, including adding a gender equity component to TANF programming [113,114], increasing access to TANF among non-caregivers, especially during recessions [117], and potentially providing multiple, smaller payments throughout the month [116]. Given the costs and bureaucratic structures necessary to implement these solutions, combined with the limited amount of dollars that states allocate toward TANF administration [17], additional studies are needed to determine the most effective and efficient strategy to reduce violence and increase cash transfers to those experiencing poverty.

To realize its potential as a violence prevention program, TANF policy-makers may consider addressing the ways in which TANF conditions create significant psychological, economic, and relationship stress among women and family systems [118,119]. Reconsidering the child support requirement and “pass-through” could be one tangible step toward preventing IPV and facilitating non-violent parental relationships. Similar to other studies [39,41], we find that multiple TANF policies act as unique barriers to IPV survivors’ access to benefits, and that these experiences were often shaped by women’s unique and overlapping social identities. Despite the wide adoption of child support protections for IPV survivors under the FVO [41], this study supports the findings of extant literature that single parents, who are also IPV survivors, face barriers to accessing the child support cooperation waiver [120] and face structural discrimination because of the very existence of this requirement [39]. Initiating a child support case appeared to cause conflict regardless of whether IPV was present and de-incentivized non-custodial parents from providing supports for and creating relationships with their children. This requirement was particularly detrimental for single mothers who sought to establish co-parenting relationships with the father of their child. Indeed, prior studies indicate that informal supports, or monies paid to custodial parents outside of the court system and in-kind supports, or items and cash provided directly to children, are often preferred and important mechanisms of support and methods of negotiating parenting relationships among couples that do not cohabitate [121]. Furthermore, fathers who are mandated to provide formal support, through court orders, for example, often pay formal supports as substitutes, rather than complements, to informal support [122]. In the case of women experiencing TANF, when fathers substitute formal supports for informal supports, women and their children receive fewer benefits owing to the requirement that they assign their rights to child support to the state. There is increasing evidence that allowing custodial, TANF recipients to keep child support payments has no effect on women’s participation in the labor force (i.e., does not detract from the welfare-to-work schema) [22,123], may reduce family conflict such as child abuse [124], and increases the likelihood that paternity will be established [125]. In light of the rising number of single-parent families in the United States [126] and TANF’s social goals, the potential benefits of maintaining a mandatory child support participation policy and providing limited monetary pass-throughs should be reconsidered. Several states, including Colorado, have had success in increasing child support pass through amounts and may provide guidance on how this might be achieved [23].

If TANF time limits continue to be implemented as a condition of TANF receipt—and many states have decreased the number of months recipients may receive TANF since 1996—efforts can be taken to address the deleterious effects on many TANF recipients, including IPV survivors [19,127]. One solution, a solely state-funded program, has been adopted by multiple states to offer support to those who reach time limits but continue to experience barriers to employment [40]. Another way to help families prepare for time limits may be to increase complementarity among formal institutions that serve overlapping populations, such as TANF, Section 8 Housing, and Food Stamps [128,129]. While programs like Section 8 Housing and Social Security Disability remain important resources, and families are often not able to save for unanticipated expenses or income losses because of income and asset limits associated with cash transfer programs [128]. This is particularly detrimental for IPV survivors whose experience of economic abuse may reduce their economic self-sufficiency and create obstacles to employment [8]. Understanding how cash transfer and support programs for families experiencing poverty restrict family earnings and savings can help identify remedies to allow families to thrive and women to live free from violence [130]. As mentioned above, TANF-income calculations could be altered to disregard income or Earned Income Tax Credits to allow families to earn more and keep more of what they earn. Secondly, it is important to explore and map out how women use multiple formal supports to participate in and exit TANF safely and with the means necessary to support their families [37,131]. In this study, women’s ingenuity and resilience, combined with formal supports, enabled them to overcome multiple issues related to childcare, transportation, and mental and physical health that disproportionately affect survivors of IPV [71] and are among the primary causes of TANF work requirement-related sanctions [57,58,132]. Without formal supports to create an enabling environment (e.g., availability of high quality childcare) for women to use TANF resources (e.g., childcare vouchers), women often had to make hard choices [133], including engaging in work without access to high quality childcare—a risk factor for poor child development and abuse [134,135]. Third, it is important to acknowledge and address how TANF policies can exacerbate women’s struggles when external formal resources are not available. In this study, when sanctions co-occurred with other TANF requirements around housing stability (i.e., the requirement to provide TANF with a stable, physical address), women fleeing IPV were particularly disadvantaged. IPV experience is common among TANF recipients [9] and is a significant barrier to employment [38], and a predictor of homelessness for women in the U.S. [136,137,138] Removing the requirement that TANF participants provide a physical home address would be one, tangible step toward making TANF more accessible to IPV survivors and other families experiencing homelessness.

TANF caseworkers could and did participate in women’s circle of formal and informal supports, a process which was more likely to occur when the participants had ongoing contact with the same caseworker. Participants were also more likely to disclose and seek support for IPV voluntarily when they had prolonged relationships that allowed them to build a rapport with caseworkers. In New York, where the TANF to poverty ratio is higher compared to Missouri and Kansas, most participants did not know their caseworker and could not build a rapport. The lack of connection with caseworkers was particularly troublesome when women sought assistance to address IPV-related issues, such as housing instability and lack of safety. Without regular contact with their TANF cash assistance caseworker, women discussed feeling deep depression and anxiety and one even mentioned regretting leaving her abuser because she faced housing instability. In this regard, increasing funding for TANF administration to hire and offer better training to TANF workers could result in a lower ratio of TANF participants to TANF caseworkers. Making it possible for caseworkers to have a manageable caseload and engage with participants could be critical to reducing violence and improving the poor economic and employment outcomes associated with involuntary removal from TANF through sanctions and time limits [118,119].

Reducing racialized stigma and discrimination associated with TANF could be another tangible step taken to address the intersecting issues of poverty, violence, and structural discrimination more frequently experienced by African American women. TANF policies created and enhanced multiple obstacles for African American women whose experiences with TANF are frequently influenced by multiple intersecting socially constructed identities defined by race, gender, and poverty. TANF stigma [63] united women’s stories and perceptions of TANF but was experienced to a greater extent and with increased consequences among African American women. African American women and their partners were distressed by the racialized nature and narratives associated with TANF receipt [65] and described being perceived as undeserving of TANF or likely to engage fraudulently with the TANF system. Studies bear out that racialized stigma has real-life policy consequences. Indeed, TANF systems have been found to disproportionately sanction African Americans [43,44], provide fewer cash benefits to African Americans living in states with higher African American populations [15], and leave African American families living with significant economic hardship [46]. Structural discrimination outside of TANF also has significant influence on the lives of African American TANF recipients. Overall, research indicates that African American women are more likely to experience the barriers to TANF participation described by women in this study, in part due to redlining into neighborhoods of concentrated disadvantage and decreased access to the informal and formal supports that make participation in TANF possible [53,56,73,139,140]. Furthermore, women in this study expressed trepidation in involving the court system in their family’s affairs either to address issues around IPV or child support. Involvement in the court system can be particularly daunting for African American women, who often lack the economic support to cut ties with the father of their child, but whose experiences with the police and justice systems leave them and their families worse off [73,140]. Continuing to incorporate a structural lens to examine differential impacts of TANF and adjacent policies by group—belonging, including race and ethnicity—will be essential to undermining the racialized stigma associated with TANF. Focusing on systems-level discrimination may also be more effective at increasing dominant group support for “reparative policies” [141,142], such that access and enjoyment of TANF may become more equitable.

In member-checking events, participants asserted that the TANF system compounds the disadvantage and relationship stressors created by multiple systems that are disproportionately experienced by African American families. Member-checking participants also indicated that racist attitudes and stereotype threat, or the fear that African Americans who access TANF are conforming to stereotypes or typical beliefs about their social group [143], compounds the shame and stigma for African American TANF participants. Given that TANF primarily serves custodial parents and their children, women bear the brunt of systems that are not structurally designed to recognize their multidimensional characteristics and provide them with appropriate services [144]. Recent studies suggest that creating social support systems may be especially important for African American women who are experiencing TANF [37].

### Strengths and Limitations

The strengths of this study include the incorporation of perspectives from a geographically diverse sample of women with years of direct experiences with TANF policies. Our community-engaged approach in which the community-based partners were involved in study protocol design, interview guide development, referrals, and member-checking of study results helped us ask meaningful and contextually relevant questions in a sensitive manner. Furthermore, our use of independent double coding data analysis supports the validity of our findings, which was further enhanced through member-checking with community-based partners and women who have experience with TANF. Our study’s limitations include the use of purposive sampling between populations that are located in cities and are already connected to formal supports, potentially limiting the transferability of findings. Only two participants had primary experience with TANF in Kansas and one of these participants was recruited via snowball sampling, suggesting additional potential bias in response and limits to transferability. We took several steps to address concerns around bias, transferability, and saturation related to the study sample, including conducting separate, confidential interviews so that women did not influence one others’ responses, creating the codebook and beginning analysis with transcripts from the three states to identify potential outlying themes or experiences, and identifying common themes that allowed for depth and nuance across participants.

## 5. Conclusions

In this study, we identified multiple ways in which TANF could be designed to reduce family violence and promote wellbeing and healthier family relationships. To improve accessibility and equity within the TANF program, this study highlights the importance of increasing TANF cash benefits, addressing racialized narratives and stigma associated with TANF, and making child support petitions an opt-in feature of TANF. In the development of policies for families experiencing poverty moving forward, our studies and others [78,113,114] suggest that cash transfer programs associated with fewer conditions could reduce the number of families experiencing poverty-related violence. Conditions appear to widen and cement inequalities [119,145] and families who experience them often have few assets upon which to rely [20,128,146,147,148,149]. Policymakers should strongly reconsider existing policies and future efforts to place work requirements and other conditions on programs that provide basic necessities, such as a Medicaid and Supplemental Nutrition Assistance Program (SNAP or Food Stamps) [150,151,152]. The American Rescue Plan of 2021, signed into law on 11 March 2011, included a temporary expansion of a monthly refundable child tax credit of up to $250 for each child 6 to 17 years old and $300 for each child under age 6. Such plans have the potential for violence reduction in that they provide monthly rather than lump sum cash benefits, which reduce economic stress and have the potential to reduce intimate partner violence [116], increase cash benefits to those experiencing deep poverty, and may disproportionately benefit communities of color [153]. While the future of TANF remains unclear, the need for public policy action to improve family wellbeing through investments in job training and educational opportunities for adults, quality care for children, and a cash transfer social safety net remains [145].

## Figures and Tables

**Table 1:** New York, Missouri, and Kansas State TANF Policies by Year.

Policy	Description	Year *	State		
			New York	Kansas	Missouri **
TANF to Poverty Ratio	The number of families on TANF for every 100 families in poverty per state	2019	42	10	11
Cash Benefits	Amount of monetary benefits per state per month allocated to a family of three with no special circumstances living in the most populated area of the state	2018	789	429	292
Lifetime Time Limits	The number of months in which an individual is eligible to receive TANF during his/her lifetime in that state	2019	60	24	45
Work—Related Sanctions	The punitive financial measures taken against an individual or family for first failing to meet TANF work requirements	2019	Benefit is reduced by the pro rata share of the noncompliant adult until compliance	Entire unit is ineligible for benefits until compliance or 3 months, whichever is longer	Benefit is reduced by 50% for at least 10 weeks. Sanction ends when participant completes 4 consecutive weeks of participation in work activities for an average of 30 h per week in the 10-week period
Child Support Sanctions	The punitive financial measures taken against an individual or family for first failing to cooperate with child support requirements	2019	The unit’s benefit is reduced by 25% until compliance	Entire unit loses benefits for 3 months	The unit’s benefit is reduced by 25% until compliance
Family Violence Option Exemptions	Work requirements exemptions for individuals who meet TANF definitions of domestic violence victims	2019	Can be exempted from work exemption	No work exemptions exist	Temporary work exemption exists while the family undergoes intensive case management
	Length of time and type of time limits extended for period in which the unit is fleeing from or receiving treatment for domestic violence or abuse	2019	Lifetime limits can be waived for at least four months and are re-evaluated at least every six months	Lifetime limits can be extended for 6 months at a time.	Lifetime limits can be extended on a case-by-case basis

* Data provided for most recent year available on the Welfare Rules Database. ** Unlike New York and Kansas, Missouri has not formally adopted the FVO, but enacted its own policies to address the needs of IPV survivors.

**Table 2:** Examples of Interview Questions.

Domain	Question Examples
Experience with TANF	Could you describe for me why you were sanctioned? Probe: Were you aware in advance that you would be sanctioned? Why or why not? How did you find out that you were sanctioned?
Relationship between TANF and mood/stress level	How did receiving TANF cash benefits affect your mood or stress level?
Relationship between TANF and intimate partner relationships	How did experiencing a sanction affect your relationship with your partner? Your interactions with your partner?
Relationship between TANF and economic well-being	How did your experience of TANF ending affect your ability to buy items that you need like transportation or groceries?

**Table 3:** Participant Characteristics by State of TANF Receipt.

	Total (n = 13)	New York (n = 5)	Missouri (n = 6)	Kansas (n = 2)
Age, mean (SD)	33 (7.0)	35 (9.2)	30.7 (4.8)	33.5 (9.2)
Race, % (n)				
Caucasian/White	23 (3)	0 (0)	17 (1)	100 (2)
African American/Black	54 (7)	80 (4)	50 (3)	0 (0)
Mixed Race	23 (3)	20 (1)	33 (2)	0 (0)
Receiving TANF Cash Assistance at Time of Interview, % (n)	69 (9)	100 (5)	67 (4)	0 (0)
Revealed IPV, % (n)	77 (10)	100 (5)	50 (3)	100 (2)
